# Explicit gender stereotypes and sexually polymorphic cognition by gender identity

**DOI:** 10.1186/s13293-025-00813-5

**Published:** 2025-12-30

**Authors:** Mina Guérin, Fanny Saulnier, Louis Cartier, Marco Hirnstein, Sébastien Hétu, Robert-Paul Juster

**Affiliations:** 1 Département de psychologie, Université de Montréal, 90 Av. Vincent-D’Indy, Outremont, Montréal, H2V 2T2 Québec Canada; 2 Centre de recherche de l’Institut universitaire en santé mentale de Montréal, 7331 rue Hochelaga, FS-145-12, Montréal, H1N 3V2, Québec Canada; 3 Département de psychiatrie et d’addictologie, Université de Montréal, 7331 Rue Hochelaga, Montréal, H1N 3V2 Québec Canada; 4 Department of Biological and Medical Psychology, University of Bergen, P.O. Box 7807, NO-5020, Bergen, Norway

**Keywords:** Sexually polymorphic cognition, Gender stereotypes, Sex differences, LGBTQ+, Gender identity, Gender diversity, Stereotype threat

## Abstract

**Background:**

Sexually polymorphic cognition (SPC) is influenced by sex differences and gender diversity. These differences have long been studied from a biological approach, but more and more studies are pointing to the importance of considering socio-culturalzgender-related factors. Several studies have shown, for example, that explicit gender stereotypes can modulate performance on cognitive tasks that are sexually dimorphic. However, no study has examined the relationship between gender stereotypes and SPC in a population that includes transgender and nonbinary (TNB) people.

**Methods:**

We recruited 488 adults who completed eight cognitive tasks measuring a range of cognitive functions during a 150-minute session. The three groups were cis women (*n* = 160), cis men (*n* = 172), and TNB people (*n* = 156). Participants were randomly assigned to three experimental conditions: a control condition and two conditions in which participants were exposed to an explicit gender stereotype prior to each task. Psychosocial data were collected using self-report questionnaires.

**Results:**

The cognitive performance of TNB people was similar to that of cis men in a judgment of line orientation task, similar to that of cis women in a fine motor skills task and superior to both cis men and cis women in a verbal learning task. Explicit gender stereotypes had no direct impact on cognitive performance. Interestingly, differences in performance between cis men, cis women and TNB people were not apparent under all experimental conditions, reflecting the inconsistency of results in the literature.

**Conclusion:**

Our results show that the inclusion of gender diverse people allows further exploration of SPC beyond “sexual dimorphisms” and that differences in cognition cannot be explained by birth-assigned sex alone. Moreover, sex/gender variations in cognition across different stereotype induction conditions highlight the lack of consistency in the literature on SPC. Future research should ascertain whether protocol features between studies can explain the variability in results and/or whether experimenters’ stereotyped beliefs inadvertnantly influence the conclusions drawn from their studies.

**Plain english summary:**

Many studies have shown that there are sex differences in cognition. Briefly, women perform better on verbal and fine motor tasks, while men perform better on spatial and mental rotation tasks. With regard to people with other gender identities and even sexual orientations other than heterosexual, the research points out that these sexual differences are reflected differently. However, more studies are needed. To understand sexual differences in cognition, several authors are interested in the role of gender stereotypes and have shown that verbalising a stereotype explicitly before a cognitive task can modify performance on this task. Our aim was therefore to determine how explicit stereotypes could influence sexual differences in cognition in a sexually and gender diverse sample. A total of 488 participants were recruited to represent different sexual orientations and gender identities. Participants completed eight cognitive tasks and several questionnaires over a period of about two hours. Participants were separated into three different experimental conditions; two in which the experimenter said before each task that it was usually better performed by men or by women, and a control condition with no stereotype induction. The results showed that gender diverse people appear to have a distinct cognitive profile from cis men and cis women. Also, explicit stereotypes did not directly affect cognitive performance in this diverse population. However, explarotory analysis reveald that gender differences in cognitive tasks varied from condition to condition, suggesting that these differences were not robust to different testing protocols.

## Highlights

Cis women perform better on fine motor and verbal learning tasks and cis men perform better on mental rotation and line orientations judgement tasks.

Transgender and nonbinary people perform better than cis men on a fine motor task, better than both cis men and cis women on a verbal learning task and better than cis women on a judgment of line orientations task.

Explicit gender stereotypes had no direct impact on cognitive performance in our sexually and gender diversified sample.

Different experimental conditions reveald variations in cognitive gender differences; the control condition reveals gender differences for mental rotation, fine motor skills and judgment of line orientation, the congruent stereotype condition reveals no gender differences, and the incongruent stereotype condition reveals gender differences for verbal fluency, verbal learning, visuospatial memory and fine motor skills.

## Background

Cognition is defined as the set of mental processes by which individuals acquire, store, process and manipulate information. Central to cognitive science, sex differences in cognition have been extensively researched [1–4]. Given our focus on both sex differences and gender diversity, we will refer to these sexual differences in cognition as *sexually polymorphic cognition* (SPC) [5] to better represent cognitive continuums. Among the SPC skills most studied are visuospatial abilities [6] and verbal abilities [7]. Fine motor skills, which are essential to the development of several cognitive processes, also show sex differences [5].

### Sex differences in cognition

#### Visuospatial abilities

Visuospatial abilities constitute a broad spectrum of skills, including spatial visualization, spatial perception, visuo-spatial judgment, mental rotation, and spatial memory [7, 8]. In general, men tend to demonstrate better visuospatial abilities than women [5, 6, 9–12]. The majority of studies concentrate on mental rotation, defined as the capacity to rotate a three-dimensional object in one’s mind, as this is the task that demonstrates the most robust sex differences [13–16]. A meta-analysis of 246 studies revealed a significant gender effect in favor of men (Cohen’s d between 0.75 and 1) in the mental rotation task of Vanderberg and Kuse [16].

Another frequently studied visuospatial skill is visuospatial judgement, which represents the ability to analyze and recognize spatial features [17]. One task used to assess this skill is the Judgment Line Orientation, as described by Benton [18]. This task demonstrates better performance in males with effect sizes lower than those reported for mental rotation (Cohen’s d values ranging from 0.65 to 0.85) [19, 20].

Furthermore, sexual polymorphisms favoring males has been observed in episodic memory of abstract images and routes, with relatively modest effect sizes (Cohen’s d ranging from 0.2 to 0.24) [10]. A frequently employed method for evaluating this capacity is the Rey-Osterrieth Complex Figure Test [21, 22]; however, some researchers have questioned the existence of sex differences in this task, with findings suggesting no significant differences [23–25]. In short, men appear to perform better on tasks involving mental rotation, visuospatial judgment, and possibly spatial memory.

#### Verbal abilities

Women show enhanced performance in the verbal domain of cognition [7]. Language is subdivided into numerous cognitive functions, supported by different neurocognitive mechanisms [26]. However, two cognitive skills involving language in particular have attracted the attention of SPC researchers: verbal memory and verbal fluency [27–29]. Verbal memory represents the ability to memorize words in short- or long-term recall. It falls under the domain of learning and memory but is linked to language. It is often measured using the California Verbal Learning Task [30], which allows for the observation of medium effect sizes (Cohen’s d of 0.48).

In contrast, verbal fluency denotes the maximum number of words produced in response to a specific prompt within a defined time frame. Women tend to perform better on verbal fluency tasks in general, though the magnitude of the sex differences depends on the nature of the task. One of the most common verbal fluency tasks is the Controlled Word Association Test [18, 31], in wich participants are asked to name as many words as possible that begin with a certain letter or to name words that belong to a certain category. Both typically yield a small female advantage with a Cohen’s d of 0.14 for letters and 0.11 for categories [26]. In the end, multiple reviews and meta-analyses provide substantial support for the proposition that women exhibit superior verbal abilities [2, 7, 26].

#### Fine motor skills

Sexual polymorphisms have also been observed in the domain of fine motor skills [32]. Fine motor skills are not regarded as cognitive abilities in the same way as memory or processing speed. However, they can interact with executive function [33–35]. Women consistently exhibit better fine motor skills compared to men from an early school age onwards [32, 36–38]. Although less studied in adults, few studies demonstrated that women exhibited stronger performance on the Perdue Pegboard, a task measuring fine motor skills [39], with a medium effect size [5].

#### Sex and gender factors

In summary, men appear to have better visuospatial abilities, whereas women are better at verbal abilities and fine motor skills. However, it is important to emphasize that these are general trends rather than absolute rules. Certain cognitive tasks may display reverse patterns, and sex differences are not consistently observed. It is more accurate to say that women are not universally superior in verbal skills, but when sex differences do appear, they typically favour women. The same applies to men: when differences in visuospatial tasks arise, they tend to favour men. However, this does not imply that men outperform women across all such tasks. These trend in sex differences are observed in a variety of cultural contexts around the world. Some authors posit that there is an innate component to SPC and place a strong emphasis on biological factors in their studies [40–42]. Researchers adopting a biological perspective maintain that sex differences in cognition are the result of inherent biological differences between the sexes that are measurable by levels of “sex” or gonadal/steroid hormones [43, 44]. Conversely, some researchers point out that focusing solely on biological factors leads to mixed results [3, 45, 46]. Focusing only on biological or only on socio-cultural factors does not reflect the complex nature of cognitive sex differences [43, 47–50].

### Gender stereotyping and cognition

An elusive socio-cultural factor increasingly studied in cognition is gender stereotypes. One method that researchers use to assess the influence of gender stereotypes on cognitive processes is stereotype induction. This involves experimental manipulation in which there is at least one experimental group and one control group. In the experimental group, the induction of a gender stereotype precedes the completion of the task.

#### Effects of stereotype induction on cognifive performance

The induction of stereotypes can influence cognitive performance in a number of ways. The best known and first documented is the *stereotype threat theory* proposed by Aronson [51] based on the first stereotype induction paradigm [52]. According to this theory, minority group members are afraid of confirming stereotypes about their group. This stereotype threat would therefore impair their performance [53]. Following this study, many researchers have used variants of this paradigm to demonstrate that gender stereotypes have an impact on cognition. Indeed, several studies have reported that women performed even worse than men on a mental rotation task when they were told that men are better [54, 55]. This effect can also be observed in men on verbal tasks, where the presence of an unfavorable stereotype negatively affects their performance [55].

Some studies have also indicated that stereotypes can, on occasion, have a beneficial impact on cognitive performance when the participant hear a positive stereotype about their group, a phenomenon that is referred to in the literature as a “boost”. For example, female participants showed enhanced performance on a mental rotation task when they were primed with a positive stereotype, specifically being informed that women typically excel in this domain [56, 57]. This effect was also observed in male participants. When a positive stereotype was induced, performance on a mental rotation task was enhanced [54]. This idea of an increase in cognitive performance when downward comparisons are made with a denigrated outgroup is sometimes referred to by the authors as a “lift” effect [58]. Whether by mentioning that the participant’s group is better or that another group is worse, the lift and boost effects can increase cognitive performance.

Finally, there is also a “stereotype reactance” effect, which has been documented to a lesser extent. This occurs when performance increases in the presence of an unfavourable stereotype or decreases in the presence of a favourable stereotype. For example, female athletes were observed to perform better at a mental rotation task in a condition where they were implicitly given a negative stereotype than in a control condition [59]. Another study showed that men with high levels of self-consciousness but low levels of gender identification performed significantly worse on a mathematical task in the condition that induced a positive stereotype [60]. It is not yet clear why this is happening. Other phenomena related to identifying with the group seem to be involved, but it could also be that the participants try to disprove stereotypes about their own group, positive or negative.

The effects of stereotype induction on cognitive performance are therefore variable and it is not possible to predict which effects will be observed. A meta-analysis of 86 studies on the impact of stereotype induction on spatial and mathematical tasks revealed that only stereotype threats (and not lift) to women exhibited a significantly different effect size from zero, while no such effect was observed in men [61]. However, there is currently no meta-analysis of this kind on other cognitive functions. Perhaps, men’s performance would change more when confronted with stereotypes in tasks where women excel. Nevertheless, it is challenging to draw direct comparisons between these studies, given the inherent variability in stereotype induction paradigms and their potential to influence the observed outcomes.

#### Variations in protocols

There are many way in wich stereotypes can be experimentaly induced. First, it can be done (1) *explicitly* by making it clear that one gender is better at the task [62] or (2) *implicitly* by suggesting that the test measures gender differences. Hausmann [54] give a good example of implicit stereotyping. They asked participants before the task to complete a questionnaire asking them to estimate the probability that the individual was male or female based on items such as “is able to understand concepts in physics”. When the stereotype is explicit, we can also decide to give an explanation to why we observe sex differences and vary this explanation [56]. Context can also be varied. For example, some authors administer the tests in groups, combining men and women together or not [55, 63]. We can also vary the medium through which the stereotype is given, for example verbalised by a person [64] or read by the participant [57]. In short, it is becoming very difficult to compare the various stereotype induction studies, and for the moment there is no consensus on how to go about it. In summary, gender stereotypes have an impact on cognitive performance and may contribute to SPC. However, studies often show contradictory results. Besides variations in stereotype induction protocols, this may be explained by the lack of consideration of other socio-cultural factors that can influence SPC.

### Sexual/gender diversity and SPC

#### Gender diversity

Gender includes several dimensions in the ways in which an individual identifies beyond their sex [65]. The sense of being a man or woman or gender diverse largely determines how people see themselves and provides an important basis for their interactions with others [66]. Gender identity can be aligned with the sex assigned at birth, which is defined as cisgender, or differ from this, as observed in the case of gender diverse people [67]. In the context of gender diversity, there are two main categories: transgender people whose gender identity is binary, men or women, and nonbinary people who identify with neither the concept of “woman” or “man” [68].

As previously discussed, sex assigned at birth represents a primary focus in the context of SPC studies. However, it is also important to consider the influence of gender-specific concepts of femininity and masculinity. For example, a transgender person’s concepts of femininity and masculinity are aligned with their gender, not necessarily their sex assigned at birth [69–71]. Consequently, numerous studies have observed that the cognitive performance of transgender people aligns more closely with their self-identified gender than with their sex assigned at birth [72–77]. More research is needed to determine how this phenomenon translates to nonbinary people [73] have yet to be represented in the SPC literature.

#### Sexual diversity

For sexual diversity, sexual orientation represents a person’s sexual, affective and emotional preference towards women, men, or other gender identities [68, 78, 79]. Sexual orientation can also influence SPC [80, 81]. Interestingly, research has demonstrated that cognitive performance is reversed in individuals who identify as sexually diverse. For example, gay men exhibit cognitive abilities more closely aligned with those of heterosexual women than with those of heterosexual men [82–84]. In summary, being part of gender or sexual diversity seems to modulate SPC; however, no study of explicit gender stereotypes and cognition has been conducted.

### Objectives and hypothesis

There are significant gaps in the existing literature on the topic of gender stereotyping and its impact on SPC. Firstly, SPC is associated with a range of cognitive abilities, including mental rotation, line orientation judgement, verbal fluency, and verbal memory. Nevertheless, the majority of studies examining the influence of gender stereotypes employ only a mental rotation task. While this task has demonstrated its efficacy, there is a need to expand the scope of cognitive tasks under examination. Secondly, gender and sexual diversity can modulate observed “sex” differences. However, no study on gender stereotypes includes these sex and gender identities. The inclusion of transgender and nonbinary (TNB) groups could increase the representativeness of these communities, which are often sidelined in studies. This inclusion could also facilitate exploration of the link between these identities and gender stereotypes.

The aim of this study is to determine whether explicit induction of gender stereotypes influences cognitive performance on a battery of tests demonstrating sexual polymorphism in a sample with a high concentration of sexually and gender diverse people. More specifically, we want to determine whether the effect of gender stereotypes varies according to gender identity. Our study also aims to confirm or refute the cognitive differences between men and women reported in the literature, and to examine the cognitive performance of gender diverse individuals. In further exploratory analyses, we also investigate whether gender-based differences in cognition are robust to different research protocols.

## Methods

This study forms part of the SEXCOG study. To date, two phases of SEXCOG have been completed. The first phase which took place between August 2021 and November 2022 concentrated on SPC and the various factors influencing it [5]. For the second phase, which took place between March 2023 and November 2024, the same protocol was employed, but with the addition of gender stereotype experimental induction. In the current study, data from these two phases are combined for analysis.

Please note throughout that when sex assigned at birth is referred to, the terms “male” and “female” are used. When referring to gender identity, the terms “men” and “women” and “gender diverse” are used. In this paper, the term gender diverse refers to people whose gender identity is different from the sex assigned at birth, including transgender and nonbinary (TNB) people. We acknowledge that the term “gender diverse” is used less by the TNB community who prefer more precise umbrella term like TNB. The term sexually diverse refers to people with a sexual orientation other than heterosexual, including, for example, gay, lesbian and bisexual people. The impact of sexual orientation on sexually polymorphic cognition is not the main focus of this paper but is discussed by another member of our team [85].

### Design and participants

**Fig. 1:**
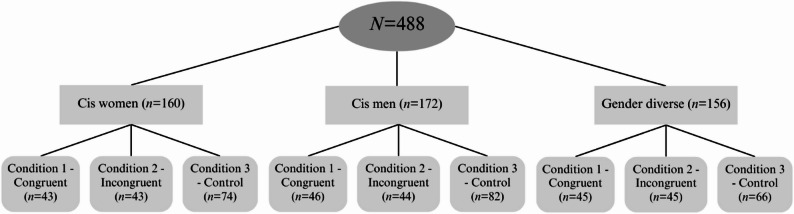
Group distribution

In the current analysis, 488 participants were recruited. Participants had to live in or be able to travel to the Greater Montreal area, and be fluent in French or English. The age eligibility requirement was 18 years and older. The age range of the final sample is 18 to 78 years old. As we wanted a diverse sample in terms of gender identity and sexual orientation, three recruitment groups were selected: cisgender and heterosexual people (*n* = 165), cisgender and sexually diverse people (*n* = 167) and gender diverse or TNB people (*n* = 156). As previously stated in the objectives, this paper places a specific emphasis on gender identity and TNB representation. Accordingly, three groups were constituted on the basis of the participants’ gender identity: *cis men* (*n* = 172), *cis women* (*n* = 160), and *gender diverse people* (*n* = 156). Note that 65% of our sample is sexually diverse. For more detailed information on group distribution, please refer to Fig. 1.

Prior to conducting our study, we engaged in a participatory project with gender diverse communities in Montreal. Our team conducted semi-structured qualitative interviews with 33 gender diverse people prior to testing. We identified health, wellness, and cognitive needs of the community and verified whether our research methodologies and variables spoke to the concerns expressed by the community that have been historically underrepresented. In the current SEXCOG study, exclusion criteria included having a diagnosis of a severe mental or physical health problem that can significantly affects cognitive abilities. This criterion was met less than five times. Participants were recruited by posting recruitment posters online, contacting organizations with a gender diverse clientele, an distributing posters in stores and in person at Gay Pride 2023 community day in Montreal.

Sample descriptive statistics are summarized in Table 1 as a function of gender identity according to demographics, socio-cultural gender variables, and general health.

**Table 1:** Descriptive statistics and groups differences according to gender

Charateristics	Sample	Cis women	Cis men	Gender diverse	*p*
***N***	488	160	172	156	-
Age, *M* (SE)^a^	29.22 (10.59)	27.36 (9.93)	31.97 (12.25)	28.09 (8.51)	< 0.001^b^
Race/ethnicity					0.265
White, *n* (%)	374 (76.80)	128 (80.50)	123 (71.51)	123 (78.85)	-
Black, *n* (%)	19 (3.90)	8 (5.03)	9 (5.23)	2 (1.28)	-
Asian, *n* (%)	21 (4.31)	6 (3.77)	7 (4.07)	8 (5.13)	-
Mixed, *n* (%)	29 (5.95)	9 (5.66)	11 (6.40)	9 (5.77)	-
Maghrebian, *n* (%)	25 (5.13)	6 (3.77)	13 (7.59)	6 (3.85)	-
Hispanic, *n* (%)	18 (3.70)	2 (1.26)	9 (5.24)	7 (4.49)	-
Indigenous, *n* (%)	1 (0.21)	0 (0.00)	0 (0.00)	1 (0.64)	-
Mother tongue					< 0.001
French, *n* (%)	351 (72.07)	131 (81.88)^c^	122 (71.35)	98 (62.82)^c^	-
English, *n* (%)	59 (12.11)	8 (5.00)^c^	18 (10.53)	33 (21.15)^c^	-
Bilingual, including French, *n* (%)	22 (4.52)	5 (3.13)	6 (3.51)	11 (7.05)	-
Others, *n* (%)	55 (11.29)	16 (10.00)	25 (14.70)	14 (8.97)	-
Language of testing					< 0.001
French, *n* (%)	398 (81.72)	145 (90.63)^c^	140 (81.40)^c^	113 (72.90)^c^	-
English, *n* (%)	89 (18.28)	15 (9.38)^c^	32 (18.60)^c^	42 (27.10)^c^	-
Hand dominance					0.490
Right, *n* (%)	441 (90.93)	146 (91.82)	151 (88.82)	144 (92.31)	-
Left, *n* (%)	44 (9.07)	13 (8.18)	19 (11.18)	12 (7.69)	-
Socioeconomics					
Education, years in school, *M*(SE)	16.53 (2.97)	16.13 (2.85)	16.79 (3.08)	16.63 (2.94)	0.111
Sex and gender					
Bith-assigned sex					< 0.001
Male, *n* (%)	210 (43.03)	0 (0.00)	172 (100.00)	38 (24.36)^c^	-
Female, *n* (%)	278 (56.97)	160 (100.00)	0 (0.00)	118 (75.64)^c^	-
Gender identity					< 0.001
Men, *n* (%)	192 (39.34)	0 (0.00)	172 (100.00)	20 (12.82)^c^	-
Women, *n* (%)	173 (35.45)	160 (100.00)	0 (0.00)	13 (8.33)^c^	-
Nonbinary, *n* (%)	123 (25.20)	0 (0.00)	0 (0.00)	123 (78.85)^c^	-
Sexual orientation					< 0.001
Heterosexual, *n* (%)	171 (35.04)	78 (48.75)	87 (50.58)	6 (3.85)^c^	-
Sexual diversity, *n* (%)	317 (64.96)	82 (51.25)	85 (49.42)	150 (96.15)^c^	-
Gender-affirming and hormonal therapy					< 0.001
None, *n* (%)	420 (86.60)	156 (98.11)	170 (99.42)	94 (60.65))^c^	-
Hormonal therapy, *n* (%)	35 (7.22)	3 (1.89)	1 (0.58)	31 (20.00))^c^	-
Gender-affirming surgery, *n* (%)	3 (0.62)	0 (0.00)	0 (0.00)	3 (1.94)	-
Both, *n* (%)	27 (5.57)	0 (0.00)	0 (0.00)	27 (17.42)^c^	-
Contraception and menstruation					
Postmenopausal, n (%)	13 (2.74)	6 (3.95)^c^	0 (0.00)	5 (3.25)^c^	0.039
Contraceptive use					< 0.001
None, *n* (%)	425 (87.09)	114 (71.25)^c^	171 (100.00)	139 (89.10)^c^	-
Contraceptive pill, *n* (%)	48 (9.84)	36 (22.50)^c^	0 (0.00)	12 (7.69)^c^	-
Hormonal IUD, *n* (%)	2 (0.41)	1 (0.63)	0 (0.00)	1 (0.64)	-
Copper IUD, *n* (%)	5 (1.02)	4 (3.13)	0 (0.00)	1 (0.64)	-
Other hormonal contraceptives, * n* (%)	8 (1.64)	5 (1.25)	0 (0.00)	3 (1.92)	-

a. *M* = mean; SE = standard error

b. Significant difference between cis men and cis women and between cis men and gender diverse people after Tukey post hoc comparisons

c. Significant difference between the frequency obtained and the frequency expected after chi-square analyses

### Procedures

**Fig. 2:**
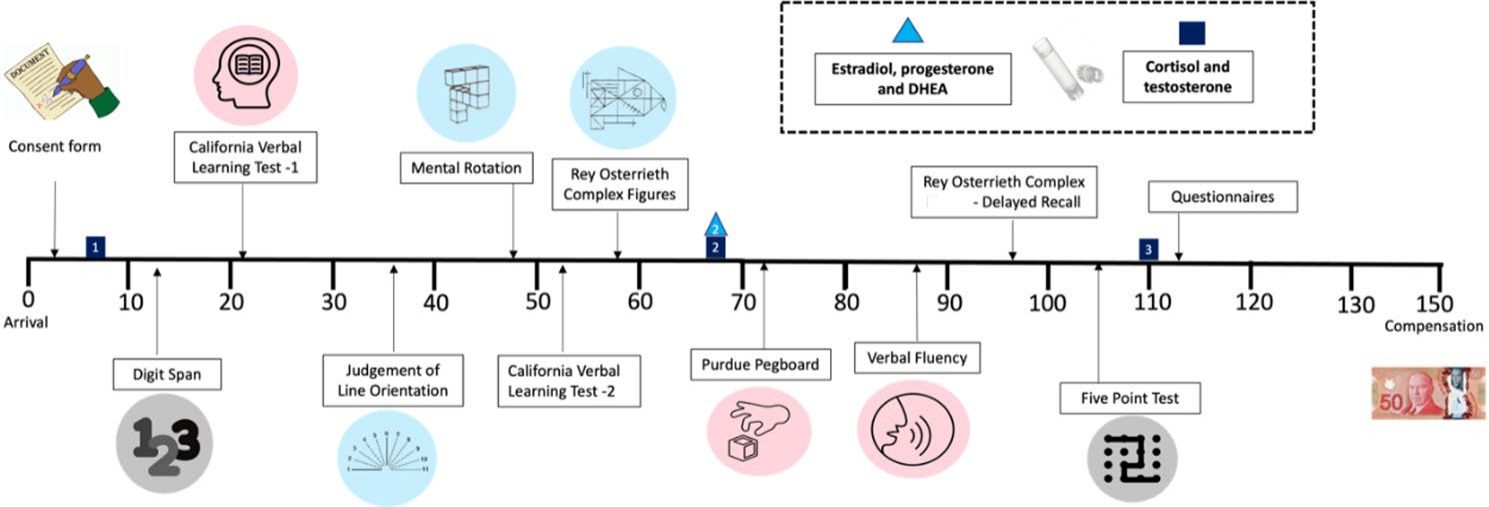
Timeline of protocol

The SEXCOG Phase 1 study is based on a published protocol paper [86]. Our study was approved by the local ethics committee. Participants visited the *Centre de Recherche de l’Institut Universitaire en Santé Mentale de Montréal* (CRIUSMM) for 2.5 h. Participants first signed a consent form and then completed eight cognitive tasks. Participants also provided saliva samples. Once this was done, participants completed an online socio-demographic questionnaire using Qualtrics and a paper questionnaire on gender beliefs. The online portion included well-validated questionnaires assessing gender identity, gender roles, and sexual orientation, as well as socioeconomic status, race/ethnicity, menstruation, contraceptive, substance use, medications, and physical and mental health. The session ended with feedback and financial compensation of 50$ (see Fig. 2). The data from the saliva samples and the gender beliefs questionnaire will be discussed further in a future dedicated paper.

### Experimental manipulation

For an overview of the distribution of participants from thesss different groups in the different conditions, please refer to Fig. 1. The participants were randomly assigned to one of three conditions: (1) a control condition, (2) a congruent experimental condition, and (3) a incongruent experimental condition. Prior to undertaking each cognitive task, participants in the experimental groups were exposed to a gender stereotype verbalized by the experimenter. The explicit stereotype induction was inspired by the studies conducted by Moè [56] and Hill [62]. The gender stereotype in question is as follows: “The objective of this test is to assess spatial abilities. Prior research has indicated that males tend to perform better than females on this particular test”.

In one experimental condition, designated the *congruent condition*, participants are presented with stereotypes that are consistent with the findings of previous studies. For example, the experimenter states that women demonstrate superior verbal memory abilities and that men exhibit greater performance in mental rotation. In the second experimental condition, referred to as the *incongruent condition*, participants received the opposite stereotype to those in the first condition. Thus, the experimenter stated that men were better at verbal memory and women were better at mental rotation. Consequently, half the participants received a stereotype that was favorable to them, and the other half received an unfavorable stereotype for each of the tests.

Among the tasks selected, we included two that did not show sexual differences as a control measure in order to show that gender stereotypes had an impact only on tasks showing sexual polymorphism. The type of stereotype induction given to participants in the congruent and incongruent conditions for these two tasks was determined randomly. The control group did not receive any stereotype induction. The type of stereotype given for each task according to experimental condition is presented in Table 2.

**Table 2:** Type of stereotypes given by task for each of the two experimental conditions

Task	Stereotype	
	Congruent condition	Incongruent condition
Digit Span	Men better	Women better
California verbal learning test	Women better	Men better
Judgment of line orientation	Men better	Women better
Mental Rotation	Men better	Women better
Rey osterrieth complex figure	Men better	Women better
Purdue pegboard	Women better	Men better
Verbal fluency	Women better	Men better
Five point test	Women better	Men better

### Cognitive mesures

Of the eight cognitive tasks chosen, three measures of skills for which women are better; namely, the California Verbal Learning Test Second-Edition, a categorical verbal fluency task and the Purdue Pegboard. Three tasks measure skills for which men are better; namely, the mental rotation, the Judgement of Line Orientation and the Rey-Osterrieth complex figure test. Two tasks show no sex difference in the literature; namely, the Digit Span and the Five Point Test.

The California Verbal Learning Test Second-Edition (CVLT- II) is a verbal learning and memory test [87]. The participant is asked to remember a certain number of words from different lists. A learning and recall phase is carried out first, followed by another delayed recall phase after a 30-minute delay. The measure reported for this task is the total of word recall for the fist list in the five trials.

To measure verbal fluency, we used a categorical verbal fluency task. Participants were given three category words and asked to generate as many words with identical or similar meanings within a time limit of 1 min per word. The categories “animal, fruit and vegetable” were used and the score represents the total number of words said for the three categories.

The Purdue Pegboard is a neuropsychological test that assesses motor skills such as dexterity and coordination [39, 88]. The participant is presented with a board featuring two parallel rows, each containing 25 holes, and is asked to insert metal pieces as quickly as possible. Four different manipulations are performed three times within a given time (30 s–1 min depending on the manipulation). The score is determined by the number of sticks inserted and the value reported in this study is a composite score of the 4 manipulations.

The Mental Rotation Task (MRT) [89] consists of line drawings representing three-dimensional objects in the form of blocks rotated to different degrees. Participants have three minutes to identify whether 20 pairs of blocks are identical or different. The score is determined by the number of correct answers.

Benton’s Judgement of Line Orientation (JLO) is a measure of visuospatial judgement that does not require motor skills [90]. This test assesses a person’s ability to match the angle and orientation of lines in space [18, 91]. The score is determined by the number of correct answers out of a total of 30.

The Rey-Osterrieth Complex Figure Test (ROCF) [21, 22] is widely used to assess visuospatial construction ability and non-verbal memory. According to the standard administration procedure [92], participants are asked to copy a figure. The figure is then hidden and, after a three-minute period, participants are asked to draw the figure as they remember it. Approximately 30 min after the immediate recall condition, participants are asked to draw the same figure again. The score was determined by the presence and correctness of 18 items out of a total of 36. The measure reported for this task is a composite score of the copy and the two recalls.

The Forward Digit Span is an attention and immediate memory test [93]. Participants are asked to repeat a sequence of numbers named by the experimenter a few seconds before. The lists become longer and longer, and the measure reported for this task is the number of digits present in the largest successful list for a maximum of 10.

The Five Point Test (FPT) is a standardized test measuring executive functioning in fluid thinking and non-verbal planning [94]. The task consists of generating drawings of different configurations in three minutes. The score is determined by the number of different configurations produced with a maximum of 25.

### Statistical analysis

Statistical analyses were performed using R software (version 4.4.1) on MacOS 14.5. Preliminary analyses were carried out to determine demographic differences between the three different groups (cis men, cis women and gender diverse) using analysis of variance (ANOVA) and χ^2^ tests. Tukey tests were used for post-hoc analyses (see Table 1). Further preliminary analyses were then conducted using the same procedure to determine whether the three stereotype-inducing conditions were demographically equivalent (see Table 3).

For the first objective, which was to determine whether stereotype induction can differentially affect cognitive performance as a function of gender identity, factorial ANOVAs were carried out. These 3 × 3 ANOVAs were used to determine how the differences between the groups changed as a function of the three experimental conditions. These factorial ANOVAs were performed for each of the eight cognitive tasks. Age was added as a covariable since significant differences between gender identities were observe (see Table 1). We decided not to include education as a corariable since no significant differences were observed between groups (see Table 1). We also decided not to include the language of administration as a covariate. Although people of gender diversity are proportionally more likely to be English speakers, we conducted additional analyses that revealed that performance on cognitive tasks did not differ according to the language of administration.

An a priori power analysis using G*power software revealed that 384 participants were needed to observe an small effect size (0.02) with a power of 0.8 and a *p* set at 0.05. The sample of 488 participants is therefore sufficient. The effect size for the power analysis was determined based on Kheloui’s review [86], Hirnstein’s meta-analysis [26], and Doyle and Voyer’s meta-analysis [61]. Simple ANOVAs were performed to analyze the simple effects of gender identity and conditions when significant. In order to correct for multiple comparisons, we used the Bonferroni correction. Thus, the critical p-value for interaction ANOVAs is set at 0.006. Tukey tests were used for post-hoc analyses.

For the second exploratory objective, which was to determine whether gender differences in cognition would persist across protocols using different administration instructions, simple ANOVAs by groups were performed for each of the tasks. These analyses were carried out separately according to the three stereotype induction conditions.

The effect sizes are reported using partial eta squared, following the parameters of Kinnear and Gray [95] for small (η²_p_ ≈ 0.01), medium (η²_p_ ≈ 0.06), and large (η²_p_ ≈ 0.14) effect sizes.

## Results

### Descriptive results

Table 1 shows the descriptive statistics according to the three groups of interest by gender identity: cis men, cis women, and gender diverse people. Cis men were significantly older than cis women (*p* < 0.001) and gender diverse people (*p* = 0.002). Differences according to mother tongue were observed, with the proportion of English speakers being greater for gender diverse people than for the other groups (*p* < 0.001). People of sexual diversity are more represented among people of gender diversity (*p* < 0.001). In addition, people taking hormone therapy or having had gender affirmation surgery and taking hormone therapy are more represented among gender diverse people (*p* < 0.001). We have included all exogenous hormone use in the hormone therapy category, apart from hormonal contraceptives. This is why some cisgender people are represented in this category, as they may, for example, take hormone therapy for menopause. No significant differences were observed according to hand dominance, education, and race/ethnicity.

Table 3 shows the descriptive statistics according to the three different experimental conditions. Participants in the incongruent stereotypes condition were significantly older than those in the control condition (*p* = 0.019). Differences according to contraceptive use were observed, with the proportion of participants taking the contraceptive pill being greater in the control condition (*p* < 0.044). No significant differences were observed according to race/ethnicity, mother tongue, hand dominance, education, birth-assigned sex, gender identity, sexual orientation, gender-affirming and hormonal therapy, and post-menopause. As significant age-related differences were observed both between groups and between different conditions, age was added as a covariate.

**Table 3:** Descriptive statistics and groups differences according to experimental conditions

Characteristics	Sample	Control	Congruent stereotypes	Incongruent stereotypes	*p*
***N***	488	222	134	132	-
Age, *M* (SE)^a^	29.22 (10.59)	27.92 (8.96)	29.19 (10.63)	31.42 (12.58)	0.019^b^
Race/ethnicity					0.703
White, *n* (%)	374 (76.80)	174 (78.38)	99 (73.88)	101 (77.10)	-
Black, *n* (%)	19 (3.90)	9 (4.05)	6 (4.48)	4 (3.05)	-
Asian, *n* (%)	21 (4.31)	6 (2.70)	7 (5.22)	8 (6.11)	-
Mixed, *n* (%)	29 (5.95)	16 (7.21)	6 (4.48)	7 (5.34)	-
Maghrebian, *n* (%)	25 (5.13)	11 (4.95)	9 (6.72)	5 (3.82)	-
Hispanic, *n* (%)	18 (3.70)	5 (2.25)	7 (5.22)	6 (4.58)	-
Indigenous, *n* (%)	1 (0.21)	1 (0.45)	0 (0.00)	0 (0.00)	-
Mother tongue					0.081
French, *n* (%)	351(72.07)	169 (76.13)	88 (65.67)	94 (71.76)	-
English, *n* (%)	59 (12.11)	22 (9.91)	16 (11.94)	21 (16.03)	-
Bilingual, including French, *n* (%)	22 (4.52)	10 (4.50)	6 (4.48)	6 (4.58)	-
Others, *n* (%)	55 (11.29)	21 (9.46)	24 (17.91)	10 (7.63)	-
Language of testing					0.098
French, *n* (%)	398 (81.72)	102 (76.69)	106 (80.30)	190 (85.59)	-
English, *n* (%)	89 (18.28)	31 (23.31)	26 (19.70)	32 (14.41)	-
Hand dominance					0.425
Right, *n* (%)	441 (90.93)	202 (90.99)	123 (93.18)	116 (88.55)	-
Left, *n* (%)	44 (9.07)	20 (9.01)	9 (6.82)	15 (11.45)	-
Socioeconomics					
Education, years in school, *M*(SE)	16.53 (2.97)	16.46 (2.72)	16.78 (2.99)	16.38 (3.33)	0.504
Sex and gender					
Bith-assigned Sex					0.730
Male, *n* (%)	210 (43.03)	123 (55.41)	80 (59.70)	75 (56.82)	-
Female, *n* (%)	278 (56.97)	99 (44.59)	54 (40.30)	57 (43.59)	-
Gender identity					0.961
Men, *n* (%)	192 (39.34)	77 (34.68)	53 (39.55)	49 (37.12)	-
Women, *n* (%)	173 (35.45)	90 (40.54)	46 (34.33)	50 (37.88)	-
Nonbinary, *n* (%)	123 (25.20)	55 (24.77)	35 (26.12)	33 (25.00)	-
Sexual orientation					0.692
Heterosexual, *n* (%)	171 (35.04)	81 (36.49)	43 (32.09)	47 (35.61)	-
Sexual diversity, *n* (%)	317 (64.96)	141 (63.51)	91 (67.91)	85 (64.39)	-
Gender-affirming and hormonal therapy					0.578
None, *n* (%)	420 (86.60)	194 (88.58)	113 (84.33)	113 (85.61)	-
Hormonal therapy, *n* (%)	35 (7.22)	17 (7.76)	10 (7.46)	8 (6.06)	-
Gender-affirming surgery, *n* (%)	3 (0.62)	1 (0.46)	1 (0.75)	1 (0.76)	-
Both, *n* (%)	27 (5.57)	7 (3.20)	10 (7.46)	10 (7.58)	-
Contraception and menstruation					
Postmenopausal, n (%)	13 (2.74)	5 (2.40)	4 (2.99)	4 (3.03)	0.923
Contraceptive use					0.044
None, *n* (%)	425 (87.09)	189 (85.14)^c^	115 (87.12)	121 (90.30)	-
Contraceptive pill, *n* (%)	48 (9.84)	28 (12.61)^c^	13 (9.85)	7 (5.22)	-
Hormonal IUD, *n* (%)	5 (1.02)	0 (0.00)	2 (1.51)	3 (2.24)	-
Copper IUD, *n* (%)	2 (0.41)	0 (0.00)	0 (0.00)	2 (1.49)	-
Other hormonal contraceptives, * n* (%)	8 (1.64)	5 (2.25)	2 (1.52)	1 (0.75)	-

(a) M = mean; SE = standard error

(b) Significant difference between the control condition and the incongruent stereotype condition after post hoc comparisons using the Tukey test

(c) Significant difference between the frequency obtained and the frequency expected after chi-square analyses

### Main analysis: cognitive performance as a function of gender identity and stereotypes

**Fig. 3:**
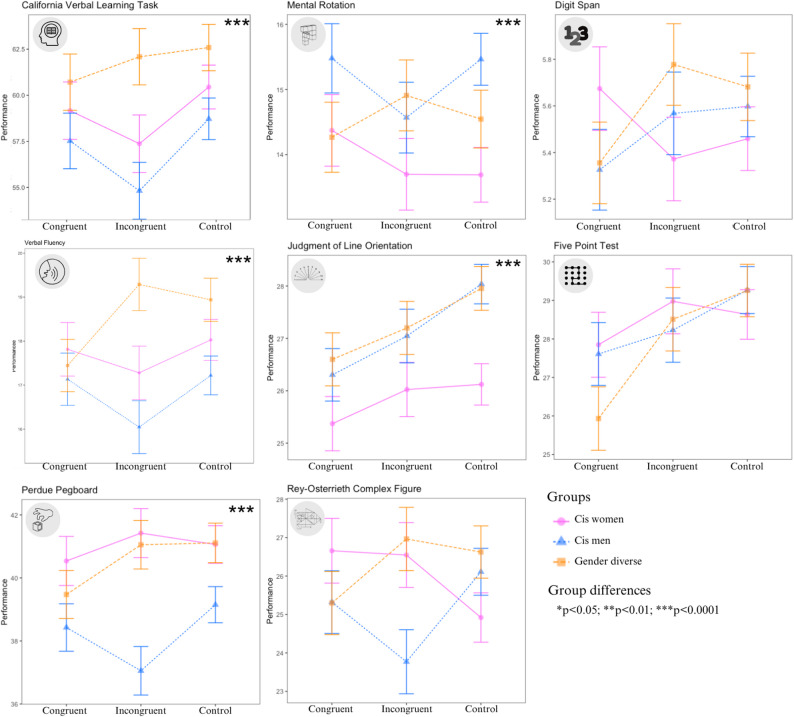
Cognitive performances in relation to gender and stereotype induction

Interaction effects for ANOVAs between groups and different conditions on cognitive performance were analyzed for each of the eight cognitive tasks. Based on preliminary analyses, age was added as a covariate. Figure 3 provides a visual representation of these results, while Table 4 shows the statistical results for single effects, interaction effects, and the covariation effect. Table 5 reports means by group and condition.

#### “Feminine” tasks

None of the three “feminine” tasks yielded significant interaction or main effects of Condition (see Table 4). There was ony a significant main effect of Groups for the California Verbal Learning Task (CVLT) and for the Perdue Pegboard with medium effect sizes. Post hoc analysis reveald that gender diverse people performed better on the CVLT than cis men (*p* = 0.003) and cis women (*p* = 0.022). For the Perdue Pegboard, gender diverse people performed better than cis men (*p* = 0.010) and cis women also performed better than cis men (*p* < 0.001). The verbal flence task showed no gender difference when the three categories (“animals”, “fruits”, “vegetables”) were put together. However, when the categories were taken separately, there was a main effect of Groups for the “fruits” category (*F*(2,478) = 4.20, *p* = 0.016, η²_p_ = 0.02). Post hoc analysis reveald that gender diverse people performed better than cis men (*p* = 0.039).

#### “Masculine” tasks

None of the three “masculine” tasks yielded significant interaction or main effects of Condition according to our critical p-value (see Table 4). For the main effect of Groups, only the Mental Rotation and the Judment of Line Orientation had significant results, with small effect sizes. Post-hoc analyses revealed that cis men performed better on the Mental Rotation than cis women (*p* < 0.001) whereas cis men (*p* < 0.001) and gender diverse people (*p* < 0.001) performed better than cis women on the Judment of Line Orientation.

#### Neutral tasks

For the two neutral tasks, no significant interaction effect or main effect of group was reveald (see Table 4). Regarding the main effects of Condition, only the Five Point Test reveald a significant and small effect. Post-hoc analyses show that participants in the control condition had better performances than those in the congruent stereotypes condition (*p* = 0.028).

**Table 4:** Interaction effect, simple effect of gender and simple effect of stereotype induction on cognitive performance

	Simple effect: group		Simple effect: condition		Interaction: groupXcondition		Covariate: age	
Task	*F*(2,478)	η²_p_	*F*(2,478)	η²_p_	*F*(4,478)	η²_p_	*F*(1,478)	η²_p_
California verbal learning task	7.48***	0.03	1.35	0.006	0.32	0.003	18.29***	0.04
Verbal fluency	2.28	< 0.001	0.82	0.003	0.99	0.008	2.39	0.005
Perdue pegborad	8.37***	0.03	1.05	< 0.001	0.65	0.006	30.78***	0.06
Mental rotation	7.96***	0.03	0.19	< 0.001	0.50	0.004	22.89***	0.05
Judgment of line orientation	8.80***	0.03	5.55**	0.02	0.39	0.002	3.10	0.002
Rey-osterrieth complexe figure	0.47	0.002	0.24	< 0.001	2.04	0.02	37.74***	0.07
Digit span	0.34	0.001	0.56	0.002	1.33	0.01	2.13	0.005
Five point test	1.29	0.005	5.08**	0.02	0.71	< 0.001	25.79***	0.05

*p < 0.05; **p < 0.01; ***p < 0.001

**Table 5:** Cognitive performances in relation to groups and stereotype induction

	Group			Condition		
**Task**	**Ciswomen**	**Cismen**	**Gender diverse**	**Control**	**Congruent**	**Incongruent**
California verbal learning task, *M* (SE)^a^	26.43 (0.57)^b^	26.17 (0.52)^c^	27.89 (0.58)^b, c^	27.14 (0.53)	26.42 (0.59)	26.47 0.58)
Verbal fluency, *M* (SE)	53.05 (1.28)	53.43 (1.16)	55.38 (1.29)	54.38 (1.19)	53.08 (1.31)	53.89 (1.25)
Perdue pegborad, *M* (SE)	38.77 (0.58)^d^	36.58 (0.52)^d, e^	38.28 (0.58)^e^	37.99 (0.55)	37.07 (0.59)	37.74 (0.57)
Mental rotation, *M* (SE)	12.80 (0.41)^f^	14.53 (0.37)^f^	13.68 (0.41)	13.73 (0.38)	14.02 (0.42)	13.66 (0.40)
Judgment of line orientation, *M* (SE)	25.21 (0.40)^g, h^	26.67 (0.36)^g^	26.70 (0.40)^h^	26.75 (0.37)^i^	25.72 (0.41)^i^	26.21 (0.39)
Rey-osterrieth complexe figure, *M* (SE)	23.50 (0.61)	23.68 (0.56)	24.02 (0.62)	23.50 (0.57)	23.54 (0.62)	24.11 (0.60)
Digit span, *M* (SE)	5.37 (0.14)	5.45 (0.13)	5.51 (0.14)	5.44 (0.13)	5.35 (0.14)	5.52 (0.14)
Five point test, *M* (SE)	26.93 (0.70)	27.51 (0.56)	26.79 (0.63)	27.60 (0.57)^j^	26.03 (0.63)^j^	27.57 (0.60)

a. *M* = mean; SE = standard error

b, c,d, e,f, g,h, i,j. Significant differences between subgroups after Tukey post-hoc tests

### Exploratory analysis: variations of gender differences in cognitive performances across different protocols

**Fig. 4:**
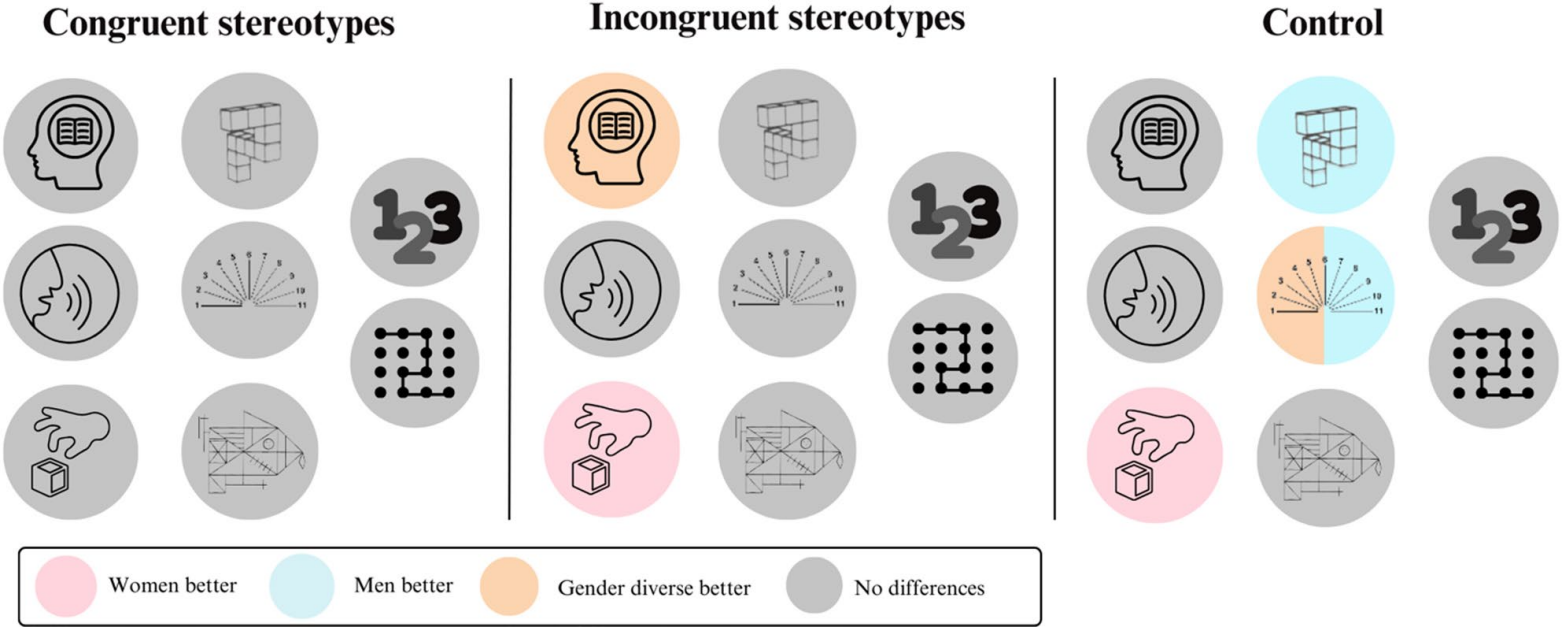
Summary of variations of gender differences in cognitive performances across different protocols

For the exploratory objective we divided our analysis by the three different conditions. Simple ANOVAs between groups were performed for each of the eight cognitive tasks. Age was also added as a covariate. Figure 4 provides a summary of the results.

#### Congruent stereotypes condition

In this condition, no significant differences were found for any of the tasks (all *F*s ≤ 2.39, all *p*s ≥ 0.097 and all η²_p_s ≤ 0.05).

#### Incongruent stereotypes condition

In this experimental condition, no significant differences were found for the Mental Rotation, the Judgment of Line Orientation, the Digit Span and the Five Point test (*F*s ≤ 0.93, *p*s ≥ 0.079 and η²_p_s ≤ 0.05). Significant differences were found for the California Verbal Leaning Task [*F*_(2,129)_ = 6.11, *p* = 0.003, η²_p_ = 0.09] for which gender diverse people had better performances than cis men (*p* = 0.002). Significant differences were also found for the Perdue Pegboard [*F*_(2,129)_ = 3.93, *p* = 0.023, η²_p_ = 0.08] for which cis women were better than cis men (*p* = 0.022).

#### Control condition

In the control condition, no significant differences were found for the California Verbal Learning Task, the Verbal Fluency, the Rey-Osterrieth Complexe Figure, the Digit Span and de Five Point Test (*F*s ≤ 2.79, *p*s ≥ 0.063 and η²_p_s ≤ 0.03). Significant difference between groups were found for the Perdue Pegboard [*F*_(2,215)_ = 3.79, *p* = 0.024, η²_p_ = 0.03]. Post-hoc Tuckey tests revealed that cis women were better than cis men (*p* = 0.0499). There was also a significant difference between groups for the Mental Rotation [*F*_(2,215)_ = 5.56, *p* = 0.005, η²_p_ = 0.06], where cis men had better performances than cis women (*p* = 0.003). The Judgment of Line Orientation also had a significant group effect [*F*_(2,215)_ = 9.88, *p* < 0.001, η²_p_ = 0.08]. Cis men had a better performance than cis women (*p* < 0.001) and gender diverse people also had a better performance than cis women (*p* < 0.001).

## Discussion

This study assessed whether an explicit gender stereotype could affect cognitive performance. To date, almost all studies of explicit gender stereotypes have not considered sexual and gender diversity at all. Our study is particularly notable for the large proportion of gender diverse or TNB people, which makes it possible to analyse their cognitive profiles with sufficient power. Contrary to our initial predictions, our results show that the interaction effect between stereotype induction and gender identity was not significant. Next, we wanted to explore whether gender differences in cognition might vary from one research protocol to another. Our results seem to show that they do, which leads us to reflect on the validity and variability of the sex differences in cognition reported in this mixed literature.

### No effect of explicit gender stereotypes on performance

Our results suggest that the explicit gender stereotypes articulated to the participants did not influence cognitive performance. Indeed, no interaction effect between gender identity and the different experimental conditions was significant for any of the eight tasks except the Rey-Osterrieth Complex figure. This is not consistent with the literature on the induction of gender stereotypes. Indeed, stereotype threat is expected to affect performance. Thus, if women are told that men are usually better at the task they are about to perform, their performance should be worse than in the control condition [54, 55]. In our study, the conditions are separated according to the initial message given to the participants during instruction. For example, in the mental rotation task, participants in the congruent condition were told that men were better at this task. In this situation, the women were under potential stereotype threat for this task, whereas the men were under potential lift or boost effects. It is difficult to determine whether gender diverse or TNB people in this condition perceived this message as a potential stereotype threat or boost/lift, so we did not have specific hypotheses for this group. We therefore expected the effect of the stereotype condition to change as a function of gender identity. The fact that stereotype induction did not exert the expected effect can be explained by various factors that we will innumerate next.

#### Sexual/gender diversity and gender stereotyping

Firstly, the most obvious hypothesis on why we did not observe interaction effects is that our sample is very diverse in terms of sexual orientation and gender identity. Indeed, it may be that TNB people are less concerned about the induction of gender stereotypes, especially the nonbinary members of this group who made up 79% of the gender diverse sample. By self-definition, they do not strictly identify with the concept of man or woman, and because there are no stereotypes about the performance of nonbinary people, they may not experience stereotype threat at all [96]. However, this does not explain why the performance of cisgender people was not affected by the stereotype conditions. We believe that this could be explained by the large proportion of sexually diverse people in our sample. In fact, 64% of cisgender people in our sample have a sexual orientation other than heterosexual.

The concept of feminity and masculinity in how sexually diverse people identify are different from those of heterosexual people [97]. For example, a lesbian woman may take on more masculine gender roles than a heterosexual woman in general. This may change her perception of the gender stereotype induced in the study. If she takes on fewer feminine gender roles, she may not be as affected when she is told that men are better at a task. Explicit gender stereotypes may therefore simply have less impact on people from the LGBTQ + community. The impact of sexual orientation on cognitive performance is discussed in another paper by our team [85] that is further complexified even further by gender role variability.

#### Methodological challenges of stereotype induction

Another hypothesis that could explain why our interaction effects were not significant is that the influence of induced stereotypes caused contradictory effects that varied from one participant to another. As described earlier, stereotype induction can cause different effects such as threat, lift or boost effects and even reactance effects (increases in performance in the presence of an unfavourable stereotype or decreases in the presence of a favourable stereotype). It is not possible to determine which effects occurred for which tasks and for which groups. It is therefore possible that certain effects were cancelled out. In addition, it has been suggested that it would be more difficult to induce stereotype threat and boost/lift effects simultaneously when the cognitive test battery included tests favouring both men and women [55]. It is therefore possible that administering eight cognitive tasks, some favouring men and others favouring women, may have limited the full effect of stereotype threat or boost/lift on performance compared with a study including only one task.

Another effect to consider is the way we framed our stereotype induction. Our stereotype induction was very explicit in that we told participants directly which gender was better at the task. Some authors opt for a more subtle way of exposing participants to the stereotype, a way that is more implicit than explicit. For example, some authors simply tell participants that the task they are about to perform involves gender differences. They argue that this is enough to induce stereotype threat. Other scholars have shown that implicitly induced stereotypes produce the largest change in cognitive performance compared to explicitly induced stereotypes [98]. Our method of induction may therefore have been too explicit to produce an effect. It may also be that the participants understood what we were doing and were more skeptical about the information we gave them.

There seem to be many possible factors that can confound the results of stereotype induction. Furthermore, the effect on cognitive performance seems to vary from study to study [61]. For these reasons, stereotype induction may not be the purest, most objective way of measuring the impact of gender stereotypes on cognition. By contrast, other authors have turned to other methods to measure the impact of gender stereotypes on cognitive performance [99]. For example, Moè measures *internalized stereotypes*, or gendered beliefs about cognitive ability. We also measured these gendered beliefs in this study, but these results will be discussed in a separate analysis as it is beyond our current scope.

### Gender differences: gender diversity adds complexity

An interesting aspect of the current analysis is the main effect of gender identity on cognitive task performance. First, we can see that some of the differences between cismen and ciswomen are consistent with the literature. Indeed, men outperformed women on all the visuospatial tasks except de Rey’s complexe figure. Women outperformed men on the fine motor task, but not on the verbal tasks. We are not surprised to see no difference in Rey’s figure, as this effect is not systematically reported in the literature. With regard to the CVLT, one of our recent papers found that performance on the CVLT was more influenced by socio-cultural gender factors than by biological sex-related factors. This could potentially explain the results observed [5]. There were no differences in verbal fluency when the three categories where taken together. This may be explained by the type of verbal fluency task we chose. Specifically, naming categories typically yields a female advantage of *d* = 0.11 according to a meta-analysis [26], which is relatively small. Thus even with a sample size of roughly 500 participants, this study may have lacked statistical power to realiably detect the female advantage. When categories were taken seperatly, results sowed that non binary people were better then men in a “fruits” category. This result has not been directly found before, but in another study where several types of category were tested and authors found that women performed better than men in a fruit category, but not in a vegetable category or in animal categories [100].

One very valuable aspect of our study is that we have many gender diverse or TNB people in our sample, which allows us to compare their performance with that of cisgender people. The results showed an advantage for gender diverse people on several tasks, including two female tasks and one male task. In fact, people in this group performed better than cis men and women on the verbal learning task, better than men on the fine motor task, and better than women on the line orientation judgement task. This is a surprising result that could potentially be explained by the inclusion of nonbinary people. Indeed, in our study, they represent approximately 80% of the gender diversity group, whereas in most studies, only transgender people are included. We wish to explore these findings further in additional analyses that will be the subject of another paper focusing on the cognitive performance of nonbinary people, their sex hormone levels, and their internalized gender roles.

Collectively, these findings show that gender identity plays a role in performance in cognitive tasks and that we cannot just look at the sex assigned at birth in SPC studies. Indeed, we need to move away from this outdated binary view of ‘sexual dimorphisms’. In neuroscience, some suggest that our brains are mosaics of typically female and typically male characteristics, and that gender categories explain only part of the variability in brain structure [101]. While there is no consensus, some of the tests used in our study are tests used in clinical evaluation, such as the California Verbal Learning task and the Judgment of Line Orientation. The norms used for these tests are gendered norms with male-female standards [102, 103]. Important clinical decisions are therefore made on the basis that there is only two cognitive profiles. In the future, it would be important to have norms for people of different gender identities.

### How sensible sexually polymorphic cognition is to the administration protocol

Our exploratory objective was to determine whether sex differences in cognition could vary from one protocol to another. As previously mentioned, the experimental manipulation did not modulate cognitive performance; however, we question whether sex differences in cognition are consistent across experimental conditions at all. Logically, if these sex differences are robust and our stereotype induction does not appear to have modulated performance, sex differences should be maintained across conditions. In fact, all the participants were recruited using the same procedures, they were tested in the same rooms, and there were no socio-demographic differences between the conditions apart from age which was controlled for (Table 3). The only notable difference was the variation in the instructions given to the participant.

Our results show the expected sex differences in the control condition, except for the verbal tasks. In the congruent stereotypes condition, surprisingly no sex differences were observed and in the incongruent stereotypes condition gender diverse people had a better performance in a few tasks. These exploratory analyses therefore suggest that there are important variations in sexual differences between the different protocols employed. This phenomenon is reflected in literature. Indeed, a recent review on SPC highlights the many contradictory studies on sex and gender differences in cognition [2].

Much of the mixed SPC literature can be explained by the small effect size of sexual differences. Although sex differences are indeed reported in the literature, the effect sizes are often small [104, 105]. It is therefore easy to lack the power to observe them, or they can easily be masked by other effects, such as stress in a testing context or the implicit biases of the experimenter. Interestingly, a meta-analysis of sex differences in verbal tasks revealed that the results tended to favor the sex of the first author of the article [26]. In this manner, if the results showed that women performed better, the first author was more likely to be a woman, and vice versa for men. Without us being aware of it, bias can be introduced into our studies, whether in the design, the administration of tasks, or even in the analysis of the data. Given that it is common for the first author to have made a significant contribution to data collection and to have been the administrator for many participants, it is also possible that the gender of the administrator may have influenced the results.

These exploratory analyses highlight a phenomenon already present in the literature: sex differences in cognition are not consistent from one study to another. Small variations in sample specifications or protocols could potentially change the conclusions. We therefore highlight the importance of transparency on the part of researchers with regard to their samples and their test protocol. More studies are needed on the impact of different assessment contexts on SPC variation.

### Strengths and limitations

This study stands out for its broad inclusion of people of sexual and gender diversity. It is one of the first studies in cognition to include so much diversity and particularly so many nonbinary people. The inclusion of these people highlights the importance of considering gender factors. We also include a comprehensive battery of SPC. Many studies on gender stereotypes and cognition use the mental rotation task, but it is much more interesting to have a more complete profile of cognitive abilities. Since the protocol incorporated eight cognitive tasks, making it highly likely that participants experienced cognitive fatigue after a certain point. However, the tasks were strategically sequenced to minimize overlap between the cognitive functions engaged, thereby reducing the risk of habituation or localized cognitive fatigue.

Another notable reservation regards the reproducibility of our results. Our sample is young, predominantly white and highly educated, so there is a possibility that these results may not be reproducible in the general population. Also, the control condition was carried out before the other two conditions. There was approximately four months between the recruitment of the last participants in the control condition and the first participants in the experimental conditions. This should be taken into account as the study was conducted in the context of the pandemic. Thus, the first phase of data collection took place in 2021 and 2022, when masks still had to be worn. The experimental phases took place in 2023, and the majority of these participants did not have to wear their masks. This may have had some effect on the cognitive tests and setting. Finally, we lack a manipulation check. We do not know whether our participants believed our stereotype inductions. We cannot ignore the possibility that this could explain why we saw no effect of stereotype conditions on performance. Future studies looking at the impact of stereotype induction on SPC performance should account for this possibility.

### Perpectives and significance

In summary, our results suggest that explicit gender stereotype induction had no effect on cognitive performance in a sample representing diversity in sexual orientation and gender identity. This could simply be explained by the fact that an experimental manipulation of this kind does not affect people from the LGBTQ community, but could also be explained by various phenomena specific to the experimental manipulation. The results highlight a unique cognitive profile of gender diverse poeple, who appear to have an advantage in verbal learning, fine motor skills, and line orientation judgement task. This demonstrates the importance of considering sex and gender factors in studies of SPC. It also highlights the importance of considering a broader, nonbinary female/male perspective. Our results also suggest that sex differences in cognition are not robust to different testing protocols and highlight the contradictions in the literature. It is essential to study experimenter bias in cognition studies and to be more critical of the sex differences observed.

## Conclusion

Overall, this study broadens our understanding of explicit gender stereotypes and sex differences in cognition. Since our explicit experimental manipulation did not directly impact performance for various reasons, it is perhaps not the best way to measure the impact of gender stereotypes on cognition and future studies could turn to other methods such as the measurement of internalized stereotypes. Our inclusive research approach highlights the importance of considering more than sex in cognitive studies. Not only do gender diverse or TNB people appear to have a distinct cognitive profile from men and women, they may also have an advantage in certain cognitive tasks. Including gender diversity and TNB communities will not only create norms of reference for this population, but also offers a unique perspective from which to study sex and gender factors.

## Data Availability

All data have been collected via participants recruited in this study. Databases wil be available upon request.
